# The complete chloroplast genome of *Actinidia fulvicoma*

**DOI:** 10.1080/23802359.2019.1691949

**Published:** 2019-11-18

**Authors:** Fuquan Zhang, Zhihao Yan, Yiqing Xu, Haifeng Lin

**Affiliations:** College of Information Science and Technology, Nanjing Forestry University, Nanjing, Jiangsu, China

**Keywords:** *Actinidia fulvicoma*, chloroplast genome, assembly, phylogeny

## Abstract

The species of genus *Actinidia* are economically and nutritionally important fruits with remarkably high vitamin C content. Here, we assembled and characterized the complete chloroplast (cp) genome sequence of *Actinidia fulvicoma* (*A. fulvicoma*) using Illumina paired-end sequencing data. The cp genome is 157,339 bp in length, including a large single-copy region (LSC) of 88,741 bp, a small single-copy region (SSC) of 20,512 bp, and a pair of 24,043 bp inverted repeat (IR) regions. A total of 131 genes, consisting of 85 protein-coding genes, 38 tRNA genes, and 8 rRNA genes, were annotated in the *A. fulvicoma* cp genome. Phylogenetic analysis confirmed the evolutionary position of *A. fulvicoma* within the genus *Actinidia*.

The classification of the genus *Actinidia* is difficult and the taxonomy of some taxa is still confusing. The species of *Actinidia* are highly variable in their vegetative structures, as well as in their flowers and fruits, which is the main reason for the difficulty in the classification of the genus. The species of *Actinidia* are particularly rich in vitamin C and vitamin K, and have been considered as medicine in traditional Chinese (Huang et al. 2013). *Actinidia fulvicoma* (*A. fulvicoma*) is mainly distributed from subtropical to tropical areas of China and Vietnam. Here, we report and characterize the complete cp genome of *A. fulvicoma*, which will pave the way for the classification of the genus *Actinidia* and provide important genomic resources for future genetic studies.

The genomic DNA was isolated from fresh leaves of *A. fulvicoma* collected from South China Botanical Garden (23°11′0.47″N, 113°21′37″E) using the DNeasy plant Mini Kit (Quiagen, Carlsbad, CA). The voucher specimen was deposited at the Herbarium of Nanjing Forestry University (accession number: 20160415AF02). After DNA extraction, an Illumina paired-end library was prepared, and then sequenced using Illumina Hiseq 2000 platform. Using *Actinidia delociosa* cp genome as a reference genome, we assembled the complete cp genome sequence of *A. fulvicoma* into a free-of-gap genome using BWA (Li 2013), AbySS (Simpson et al. 2009), CD-Hit (Li and Godzik 2006), and MacVector v17.0.7 from Illumina sequencing data, and annotated it using Plastid Genome Annotator (PGA) (Qu et al. 2019). The annotated cp genome of *A. fulvicoma* was then submitted to GenBank with accession number MN540960. The circular genome of *A. fulvicoma* was 157,339 bp in length, with a large single-copy (LSC) region of 88,741 bp, a small single-copy (SSC) region of 20,512 bp, and a pair of inverted repeat (IR) regions of 24,043 bp. The overall GC content of the whole cp genome was 37.17%, while the corresponding GC content of the IR regions was 42.92%, which was higher than that of LSC (35.46%) and SSC (31.1%). A total of 131 genes were annotated in *A. setosa* cp genome, including 85 protein-coding genes, 38 tRNA genes, and 8 rRNA genes. Among the 131 genes, most of them only contain one single copy, while 15 genes contain two copies (*ndhB*, *rps7*, *ycf2*, *ycf15*, *rrn4.5*, *rrn5*, *rrn16*, *rrn23*, *trnA*-*UGC*, *trnfM*-*CAU*, *trnH*-*GUG*, *trnL*-*CAA*, *trnN*-*GUU*, *trnR*-*ACG*, and *trnV*-*GAC*), and two genes (*rps12* and *trnI-CAU*) were found to contain three and four copies, respectively.

To further clarify the phylogenetic position of *A. fulvicoma*, a neighbor-joining (NJ) tree was constructed based on 76 conserved protein-coding genes of 23 other plant cp genomes using MEGA 6.0 with 1000 bootstrap replicates (Tamura et al. 2013). As shown in Figure 1, we found that *A. fulvicoma* was clustered with other two *Actinidia* species with 100% bootstrap values. Additionally, *A. fulvicoma* was highly supported to evolutionarily close to *Nicotiana tabacum*, *Capsicum annuum*, and *Solanum lycopersicum* in the family Solanaceae.

**Figure 1.**
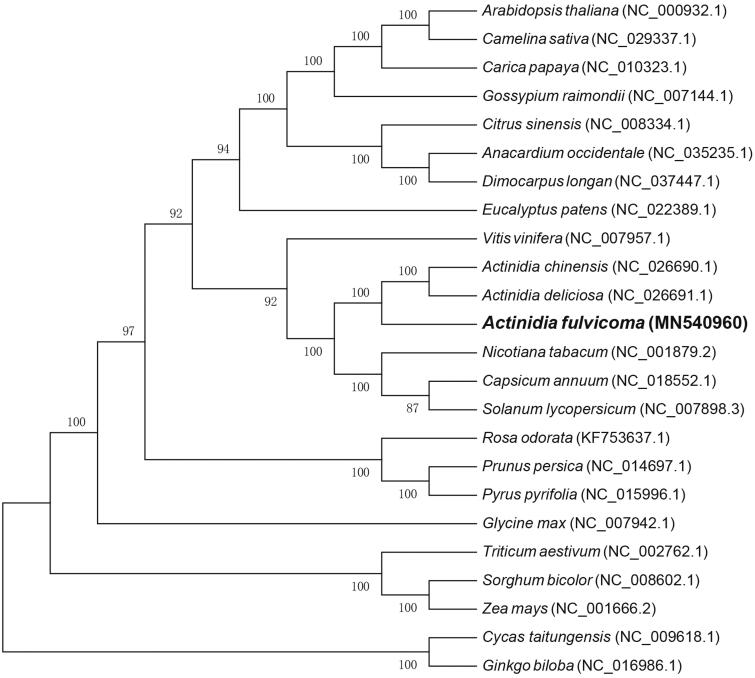
The Neighbor-Joining phylogenetic tree of 24 plant chloroplast genomes based on 76 conserved protein-coding genes. Bootstrap values are listed for each node. GenBank accession numbers are listed right to the scientific names.

## References

[CIT0001] Huang SX, Ding J, Deng DJ, Tang W, Sun HH, Liu DY, Zhang L, Niu XL, Zhang X, Meng M, et al. 2013 Draft genome of the kiwifruit *Actinidia chinensis*. Nat Commun. 4:2640. 24136039 10.1038/ncomms3640 PMC4089393

[CIT0002] Li H 2013 Aligning sequence reads, clone sequences and assembly contigs with BWA-MEM. arXiv. 1303:3997.

[CIT0003] Li W, Godzik A 2006 Cd-hit: a fast program for clustering and comparing large sets of protein or nucleotide sequences. Bioinformatics. 22(13):1658. 16731699 10.1093/bioinformatics/btl158

[CIT0004] Qu XJ, Moore MJ, Li DZ, Yi TS 2019 PGA: a software package for rapid, accurate, and flexible batch annotation of plastomes. Plant Methods. 15(1):50. 31139240 10.1186/s13007-019-0435-7 PMC6528300

[CIT0005] Simpson JT, Wong K, Jackman SD, Schein JE, Jones SJ, Birol I 2009 ABySS: a parallel assembler for short read sequence data. Genome Research. 19(6):1117. 19251739 10.1101/gr.089532.108 PMC2694472

[CIT0006] Tamura K, Stecher G, Peterson D, Filipski A, Kumar S 2013 MEGA6: molecular evolutionary genetics analysis version 6.0. Mol Biol Evol. 30(12):2725–2729. 24132122 10.1093/molbev/mst197 PMC3840312

